# Impact of serum omentin-1 levels on cardiac prognosis in patients with heart failure

**DOI:** 10.1186/1475-2840-13-84

**Published:** 2014-04-23

**Authors:** Taro Narumi, Tetsu Watanabe, Shinpei Kadowaki, Daisuke Kinoshita, Miyuki Yokoyama, Yuki Honda, Yoichiro Otaki, Satoshi Nishiyama, Hiroki Takahashi, Takanori Arimoto, Tetsuro Shishido, Takuya Miyamoto, Isao Kubota

**Affiliations:** 1 Department of Cardiology, Pulmonology, and Nephrology, Yamagata University School of Medicine, 2-2-2 Iida-nishi, Yamagata 990-9585, Japan

**Keywords:** Omentin-1, Heart failure, Prognosis

## Abstract

**Background:**

Various adipokines are reported to be associated with the development of heart failure (HF) through insulin resistance and chronic inflammation. Omentin-1 is a novel adipokine and is associated with incident coronary artery disease. However, it remains unclear whether serum omentin-1 levels are associated with cardiac prognosis in patients with HF.

**Methods:**

We measured serum omentin-1 levels at admission in 136 consecutive patients with HF, and 20 control subjects without signs of significant heart disease. We prospectively followed patients with HF to endpoints of cardiac death or re-hospitalization for worsening HF.

**Results:**

Serum omentin-1 levels were markedly lower in HF patients with cardiac events compared with to without. The patients who were in New York Heart Association (NYHA) functional class IV showed significantly lower serum omentin-1 levels compared to those in class II and III, whereas serum omentin-1 levels did not correlate with serum brain natriuretic peptide levels (r = 0.217, P = 0.011). We divided the HF patients into three groups based on the tertiles of serum omentin-1 level (low T1, middle T2, and high T3). Multivariate Cox hazard analysis showed that the lowest serum omentin-1 level (T1) was independently associated with cardiac events after adjustment for confounding factors (hazard ratio 5.78, 95% confidence interval 1.20-12.79). We divided the HF patients into two groups according to the median serum omentin-1 levels. Kaplan-Meier analysis revealed that the patients with low serum omentin-1 levels had a higher risk of cardiac events compared with those with high serum omentin-1 levels (log-rank test p < 0.001).

**Conclusion:**

Decreased serum omentin-1 levels were associated with a poor cardiac outcome in patients with HF.

## Background

Heart failure (HF) remain a major cause of death worldwide and has a poor prognosis despite advances in treatment [1]. Adipocytokines, such as tumor necrosis factor-alpha, interleukin-6, and plasminogen activator inhibitor-1, play a crucial role in the development of cardiovascular diseases through insulin resistance and chronic inflammation [2-5]. Adipokines, such as adiponectin, are also reported to have anti-inflammatory, anti-oxidant, and anti-apoptotic properties, and are decreased in patients with cardiovascular disease [6-9]. There has been a move to clarify the causal relationship between various adipokines and cardiovascular disease [10,11].

Omentin-1 is a novel adipokine whose serum levels are decreased in obese individuals, and is associated with insulin resistance [12-16]. Omentin-1 has been suggested to play a beneficial role in preventing atherosclerosis [17,18], however, it remains unclear whether serum omentin-1 levels are associated with clinical outcome in patients with HF.

The purpose of this study was to clarify the impact of serum omentin-1 levels on cardiac prognosis in patients with HF.

## Methods

### Study population

We enrolled 136 consecutive patients who were admitted to the Yamagata University Hospital for treatment of worsening HF, diagnosis and pathophysiological investigations, or for therapeutic evaluation of HF. We also enrolled 20 control subjects without signs of significant heart disease.

A diagnosis of HF was based on a history of dyspnea and symptoms of exercise intolerance followed by pulmonary congestion, pleural effusion, or left ventricular enlargement by chest X-ray or echocardiography [19,20]. Control subjects were excluded if they had significant coronary artery disease, systolic and diastolic dysfunction, valvular heart disease, or myocardial hypertrophy on echocardiography [21]. All patients gave written informed consent prior to their participation, and the protocol was approved by the institution’s Human Investigation Committee. The procedures were performed in accordance with the Helsinki Declaration.

### Measurement of serum omentin-1 and brain natriuretic peptide levels

Blood samples were drawn at admission and centrifuged at 2,500 g for 15 minutes at 4°C within 30 minutes of collection. The serum was stored at -80°C until analysis. Serum omentin-1 concentrations were measured with a sandwich enzyme-linked immunosorbent assay (ELISA, Immuno-Biological Laboratories CO., Ltd., Gunma, Japan), according to the manufacturer’s instructions [22,23]. The serum omentin-1 levels were measured in duplicate by an investigator unaware of the associated patients’ characteristics. Serum brain natriuretic peptide (BNP) concentrations were measured using a commercially available specific radio-immuno assay for human BNP (Shiono RIA BNP assay kit, Shionogi & Co., Ltd., Tokyo, Japan) [24].

### Endpoints and follow-up

The patients were prospectively followed for a median duration of 399 ± 378 days. The end points were cardiac death, including death due to progressive HF, myocardial infarction, stroke and sudden cardiac death, and re-hospitalization for worsening HF. Sudden cardiac death was defined as death without definite premonitory symptoms or signs, and was confirmed by the attending physician. Two cardiologists who were blinded to the blood biomarker data reviewed the medical records and conducted telephone interviews to survey the incidence of cardiovascular events.

### Statistical analysis

Data are presented as the mean ± standard deviation (SD). The Mann–Whitney U-test was used when the data were not distributed normally. If the data were not distributed normally, they were presented as medians with an interquartile range. The unpaired Student’s t-test and the chi-square test were used for comparisons of continuous and categorical variables, respectively. Comparison of data among three groups was performed by the Kruskal-Wallis test. Uni- and multivariate analyses with Cox proportional hazard regression were used to determine significant predictors of cardiovascular events. Cumulative overall and event-free survival rates were computed using the Kaplan-Meier method and were compared using the log-rank test. We calculated the net reclassification improvement (NRI) and the integrated discrimination improvement (IDI) to measure the quantity of improvement for the correct reclassification and sensitivity according to the addition of serum omentin-1 levels to the prediction model [25]. NRI and IDI are new statistical measures to assess and quantify the improvement in risk prediction offered by a new marker. A *P* value < 0.05 was considered statistically significant. All statistical analyses were performed with a standard statistical program package (JMP version 10; SAS Institute, Cary, North Carolina, USA), and the R-3.0.2 with additional packages (Rcmdr, Epi, pROC, and PredictABEL).

## Results

### Comparison between patients with and without heart failure

The patients with HF had a lower BMI and left ventricular ejection fraction, and lower serum total cholesterol, triglyceride levels, and higher serum BNP levels compared with control subjects (Table 1).

**Table 1:** Baseline clinical characteristics

	**Control (n=20)**	**Heart failure (n=136)**	***P *** **value**
Age, years	65 ± 16	72 ± 12	0.034
Male, n (%)	11 (55)	76 (56)	0.941
NYHA functional class, II/III/IV	-	71/46/19	-
Etiology, n (%)			-
Dilated cardiomyopathy	-	29 (21)	
Valvular heart disease	-	38 (28)	
Ischemic heart disease	-	30 (22)	
Hypertensive heart disease	-	14 (10)	
Hypertrophic cardiomyopathy	-	9 (7)	
Others	-	16 (12)	
Presentation profile			
Systolic pressure, mmHg	119 ± 22	117 ± 18	0.772
Diastolic pressure, mmHg	77 ± 10	74 ± 10	0.209
Body mass index, kg/m^2^	23.3 ± 3.4	21.7 ± 3.9	0.049
eGFR, ml/min/1.73m^2^	70 ± 24	62 ± 26	0.197
Blood biomarkers			
Albumin, g/dl	3.8 ± 0.5	3.5 ± 0.6	0.091
Total cholesterol, mg/dl	185 ± 34	166 ± 39	0.042
Triglyceride, mg/dl	143 ± 96	91 ± 47	<0.001
LDLc, mg/dl	111 ± 28	100 ± 37	0.213
HDLc, mg/dl	52 ± 15	53 ± 23	0.831
hsCRP, mg/dl (IQR)	0.121 (0.040-0.551)	0.198 (0.064-0.606)	0.279
BNP, pg/ml (IQR)	82 (50–152)	484 (215–1251)	<0.001
Omentin-1, ng/ml (IQR)	494 (351–630)	305 (35–473)	0.035
Echocardiographic data			
LV end-diastolic diameter, mm	53 ± 8	55 ± 11	0.438
LV ejection fraction, %	65 ± 9	50 ± 18	<0.001
Medications, n (%)			
ACE inhibitors and/or ARBs	15 (75)	102 (75)	0.999
β blockers	15 (75)	103 (76)	0.943
Statins	10 (50)	51 (38)	0.321
Ca channel blockers	5 (25)	37 (27)	0.778

Data are presented as mean±SD or % unless otherwise indicated; ACE, angiotensin-converting enzyme; ARB, angiotensin receptor blocker; BNP, brain natriuretic peptide; BUN, Blood urea nitrogen; eGFR, estimated glomerular filtration rate; HDLc, high density lipoprotein cholesterol; hsCRP, high-sensitivity C-reactive protein; IQR, interquartile range; LDLc, low density lipoprotein cholesterol; LV, left ventricular; NYHA, New York Heart Association.

### Comparison between HF patients with and without cardiac events

**Figure 1:**
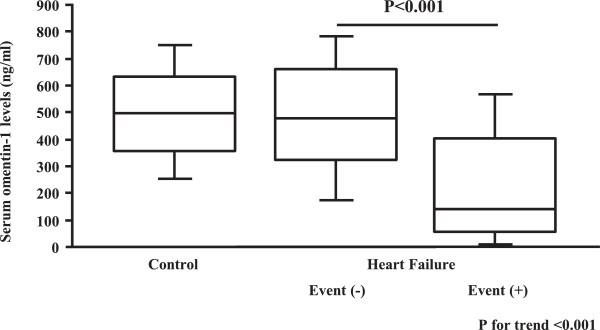
**Comparisons of serum omentin-1 levels between control subjects and HF patients with or without cardiac events.** HF patients with cardiac events showed markedly lower serum omentin-1 levels compared with those without (p < 0.001). HF, heart failure.

There were 59 cardiac events including 17 deaths and 32 re-hospitalizations in patients with HF during the follow-up period (Table 2). The patients who experienced cardiac events were in a more severe New York Heart Association (NYHA) functional class, and had a lower estimated glomerular filtration rate, lower left ventricular ejection fraction, higher left ventricular end-diastolic diameter, and higher serum BNP levels compared with those who did not. Moreover, patients with cardiac events showed markedly lower serum omentin-1 levels compared with those without (Figure 1). There were no significant differences in etiologies of HF between patients with and without cardiac events (Table 2).

**Table 2:** Comparison of patients with or without cardiac event

	**Event (-) (n=77)**	**Event (+) (n=59)**	***P *** **value**
Age, years	71 ± 10	72 ± 14	0.687
Male, n (%)	40 (52)	36 (61)	0.480
NYHA functional class, II/III/IV	49/21/6	22/25/13	0.005
Etiology, n (%)			0.348
Dilated cardiomyopathy	12 (16)	17 (29)	-
Valvular heart disease	24 (31)	14 (24)	-
Ischemic heart disease	17 (22)	13 (22)	-
Hypertensive heart disease	8 (10)	6 (10)	-
Hypertrophic cardiomyopathy	6 (8)	3 (5)	-
Others	10 (13)	6 (10)	-
Presentation profile			
Systolic pressure, mmHg	116 ± 18	118 ± 19	0.598
Diastolic pressure, mmHg	73 ± 9	74 ± 11	0.780
Body mass index, kg/m^2^	22.0 ± 4.4	21.4 ± 3.3	0.413
eGFR, ml/min/1.73m^2^	66 ± 27	57 ± 24	0.046
Blood biomarkers			
Albumin, g/dl	3.5 ± 0.6	3.5 ± 0.6	0.539
Total cholesterol, mg/dl	169 ± 37	163 ± 42	0.398
Triglyceride, mg/dl	97 ± 48	84 ± 45	0.132
LDLc, mg/dl	101 ± 37	99 ± 37	0.731
HDLc, mg/dl	55 ± 29	52 ± 12	0.437
hsCRP, mg/dl (IQR)	0.174 (0.058-0.330)	0.267 (0.073-0.722)	0.308
BNP, pg/ml (IQR)	453 (248–1249)	512 (169–1255)	0.049
Omentin-1, ng/ml (IQR)	479 (323–661)	139 (57–402)	<0.001
Echocardiographic data			
LV end-diastolic diameter, mm	53 ± 10	57 ± 10	0.011
LV ejection fraction, %	56 ± 17	45 ± 16	<0.001
Medications, n (%)			
ACE inhibitors and/or ARBs	54 (70)	48 (81)	0.134
β blockers	52 (68)	51 (86)	0.029
Statins	23 (30)	28 (47)	0.460
Ca channel blockers	29 (3)	8 (14)	0.964

Data are presented as mean±SD or % unless otherwise indicated; ACE, angiotensin-converting enzyme; ARB, angiotensin receptor blocker; BNP, brain natriuretic peptide; BUN, Blood urea nitrogen; eGFR, estimated glomerular filtration rate; HDLc, high density lipoprotein cholesterol; hsCRP, high-sensitivity C-reactive protein; IQR, interquartile range; LDLc, low density lipoprotein cholesterol; LV, left ventricular; NYHA, New York Heart Association.

### Serum omentin-1 levels and HF severity

**Figure 2:**
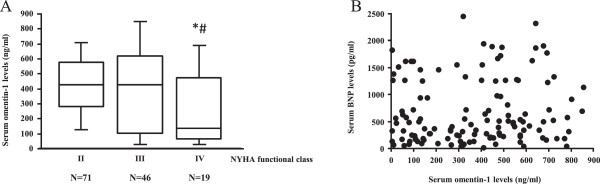
**Serum omentin-1 levels and heart failure severity. A**. The patients who were in NYHA functional class IV showed significantly lower serum omentin-1 levels compared to those in class II and III (*P = 0.029 vs. class II and #P = 0.041 vs. class III, Figure 2A). (The number of patients; II = 71, III = 46, IV = 19) **B**. The association between serum omentin-1 levels and serum BNP levels. There was no relationship between the serum omentin-1 levels and the serum BNP levels (r = 0.217). BNP, brain natriuretic peptide; NYHA, New York Heart Association.

The patients who were in NYHA functional class IV showed significantly lower serum omentin-1 levels compared to those in class II and III (P = 0.029 vs. class II and P = 0.041 vs. class III, Figure 2A). On the other hand, serum omentin-1 levels were not significantly different between the patients who were in NYHA functional class II and III (P = 0.582). Furthermore, there was no relationship between the serum omentin-1 levels and the serum BNP levels (r = 0.217, Figure 2B).

### Association between serum omentin-1 levels and cardiac events

**Figure 3:**
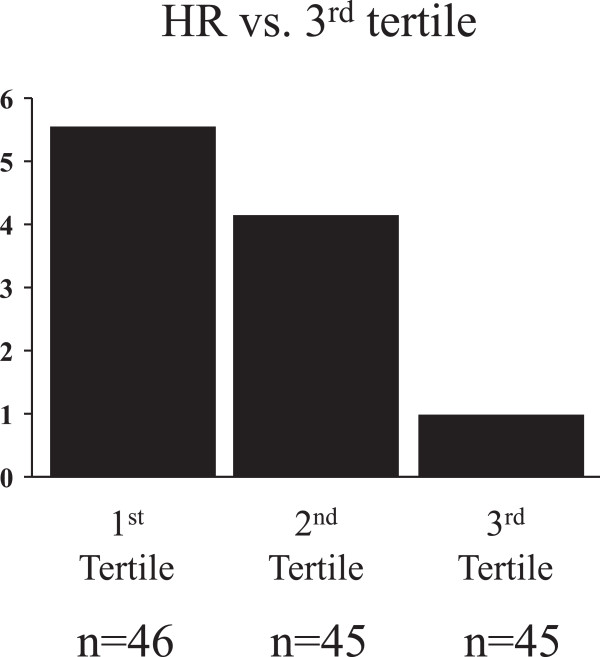
**Hazard ratio of the tertiles of omentin-1 levels for cardiac events after adjustment of age, gender, body mass index, NYHA functional class, left ventricular ejection fraction, serum triglycerides, serum HDLc levels, and serum BNP levels.** BNP, brain natriuretic peptide; HDLc, high density lipoprotein cholesterol; NYHA, New York Heart Association.

**Figure 4:**
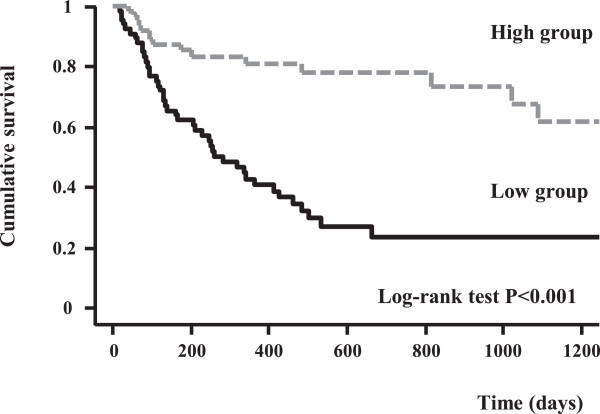
**Kaplan-Meier analysis.** The patients with low serum omentin-1 levels had a higher risk of cardiac events compared to those with high serum omentin-1 levels (log-rank test p < 0.001).

We divided patients with HF into three groups according to the tertiles of serum omentin-1 levels. Multivariate Cox hazard analysis showed that the lowest serum omentin-1 levels (T1) were independently associated with cardiac events after adjustment for age, gender, NYHA functional class, left ventricular ejection fraction, and serum brain natriuretic peptide levels (hazard ratio 5.65, 95% confidence interval 2.61-12.20; Figure 3, Table 3). We divided the patients into two groups according to the median serum omentin-1 levels. Kaplan-Meier analysis revealed that the patients with low serum omentin-1 levels had a higher risk of cardiac events compared to those with high serum omentin-1 levels (log-rank test p < 0.001, Figure 4).

**Table 3:** Univariate and multivariate analyses for cardiac events

	**Univariate Analysis**			**Multivariate Analysis**		
	**HR**	**95% CI**	***P *** **value**	Adjusted HR*	**95% CI**	***P *** **value**
Omentin-1						
T3	1	Reference	Reference	1	Reference	Reference
T2	5.56	2.85-10.87	<0.001	4.15	2.03-8.47	<0.001
T1	6.29	1.30-13.06	<0.001	5.65	2.61-12.20	<0.001

*Adjusted HR after adjustment for age, gender, body mass index, NYHA functional class, left ventricular ejection fraction, serum triglycerides, serum HDLc levels, and serum BNP levels.

BNP, brain natriuretic peptide; CI, confidence interval; HDLc, high density lipoprotein cholesterol; HR, hazard ratio; NYHA, New York Heart Association; SD, standard deviation.

### Net reclassification improvement and integrated discrimination improvement

To measure the quantity of improvement for the correct reclassification and sensitivity according to the addition of serum omentin-1 levels to the prediction model, we calculated the NRI and the IDI. The inclusion of serum omentin-1 levels in the prediction model (includes age, gender, NYHA functional class, left ventricular ejection fraction, and serum BNP levels) for the prediction of cardiac events, improved the NRI and IDI values, suggesting effective reclassification and discrimination (Table 4).

**Table 4:** Statistics for model fit and improvement with addition of serum omentin-1 level predicted on the prediction of cardiac events

	**Prediction model**	**Prediction model + omentin-1**	**P value**
NRI (95% CI)	Reference	0.375 (0.129-0.620)	0.002
IDI (95% CI)	Reference	0.149 (0.087-0.211)	<0.001

Prediction model includes age, gender, NYHA functional class, left ventricular ejection fraction, and serum BNP levels.

BNP, brain natriuretic peptide; CI, confidence interval; IDI, integrated discrimination improvement; NRI, net reclassification improvement; NYHA, New York Heart Association.

## Discussion

The present study demonstrated that decreased serum omentin-1 levels predicted cardiac events in patients with HF. Serum omentin-1 level appears to be a novel prognostic marker for the risk stratification of patients with HF.

Various types of adipocytokines are reported to be a predictor of unfavorable cardiac outcomes in patients with HF [26]. In addition to their roles as predictors of cardiac outcome, a variety of adipocytokines have been associated with the development of HF through insulin resistance and chronic inflammation [14,27-29]. Serum adiponectin levels are reported to be correlated with BNP levels, and are associated with HF severity and unfavorable outcomes in patients with HF [30,31]. Adiponectin has been suggested to play a role in the prevention of cardiovascular diseases via its anti-inflammatory, anti-oxidant, and anti-apoptotic properties [6-9]. Recently, reports have shown several adipokines to have beneficial effects on cardiovascular diseases [32-34]. However, the precise role of these adipokines remains unclear.

Omentin-1 is a 38 kDa novel adipokine identified in 2004 from visceral adipose tissue [12,13]. Shibata et al. reported that decreased plasma omentin-1 levels predict the prevalence of coronary artery disease [18]. Yang et al. reported that omentin-1 enhances insulin-stimulated glucose uptake in human adipocytes and may regulate insulin sensitivity [13]. Yamawaki et al. reported that omentin-1 modulates vascular function and attenuates cyclooxygenase-2 expression and c-jun N-terminal kinase (JNK) activation in cytokine-stimulated endothelial cells [35,36]. These studies all suggest that omentin-1 may improve insulin resistance and suppress vascular inflammation. Interestingly, Pan et al. suggested that omentin-1 expression and production are decreased with elevated inflammatory adipokines, such as tumor necrosis factor-alpha and interleukin-6, in patients with impaired glucose intolerance and newly diagnosed type 2 diabetes mellitus [37].

Unlike to adiponectin, serum omentin-1 was reported to decrease with chronic inflammation and oxidative stress in patients with HF. The bioactivity of omentin-1 appears multifaceted and remains to be fully defined. The present study showed no correlation between serum omentin-1 and BNP levels unlike adiponectin [30], suggesting that these markers indicate different features of the pathophysiological process of HF. Serum omentin-1 levels may represent a promising biomarker for cardiac prognosis, irrespective of serum BNP levels. The inclusion of serum omentin-1 levels in the prediction model (includes age, gender, NYHA functional class, left ventricular ejection fraction, and serum BNP levels) for the prediction of cardiac events, improved the NRI and IDI values, suggesting effective reclassification and discrimination.

The present study has certain limitations. Firstly, the sample size was relatively small and it was a single center study. Nonetheless, there was a significant relationship between serum omentin-1 levels and cardiac events. In addition, the inclusion of serum omentin-1 levels in the prediction model with conventional risk factors, including serum BNP levels, for the prediction of cardiac events, improved the NRI and IDI values. Secondly, there were no data for other adipocytokines. Further study is needed to clarify the association between serum omentin-1 and other adipocytokines in a large HF population.

In conclusion, decreased serum omentin-1 levels were associated with cardiac events in patients with HF, irrespective of serum BNP levels. Serum omentin-1 level appears to represent a novel prognostic marker for the risk stratification of patients with HF.

## Abbreviations

BMI: Body mass index; BNP: Brain natriuretic peptide; eGFR: Estimated glomerular filtration rate; ELISA: Sandwich enzyme-linked immunosorbent assay; HF: Heart failure; IDI: Integrated discrimination improvement; NRI: Net reclassification improvement; NYHA: New York heart association; SD: Standard deviation.

## Competing interests

The authors report that there is no duality of interest associated with this manuscript.

## Authors’ contributions

TN, TW and IK contributed to discussions about study design and data analyses. SK, DK, MY, YO, and YH conceived and carried out experiments. TN and TW participated in the interpretation of the results and the writing of the manuscript. SN, HT, TA, TS, and TM helped with data collection. All authors have read and approved the final manuscript.
